# Additive Impacts of Liveweight and Body Condition Score at Breeding on the Reproductive Performance of Merino and Non-Merino Ewe Lambs

**DOI:** 10.3390/ani14060867

**Published:** 2024-03-12

**Authors:** Andrew N. Thompson, Mark B. Ferguson, Gavin A. Kearney, Andrew J. Kennedy, Lyndon J. Kubeil, Claire A. Macleay, Cesar A. Rosales-Nieto, Beth L. Paganoni, Jason P. Trompf

**Affiliations:** 1 Centre for Animal Production and Health, Murdoch University, Murdoch, WA 6150, Australia; 2 36 Payne Road, Hamilton, VIC 3300, Australia; 3 Agriculture Victoria, Hamilton, VIC 3300, Australia; 4 Agriculture Victoria, Benalla, VIC 3672, Australia; 5 Department of Primary Industries and Regional Development, Bunbury, WA 6230, Australia; 6 UWA Institute of Agriculture, University of Western Australia, Crawley, WA 6009, Australia; 7 J.T Agri Source Pty Ltd., Mill Park, VIC 3082, Australia

**Keywords:** ewe lamb, liveweight, body condition score, fertility, reproductive rate, within flock selection

## Abstract

**Simple Summary:**

Fatness is linked to reproductive performance in sheep, and it is realistic to expect that higher body condition scores at breeding would have positive effects on the reproductive rate of ewe lambs, over and above liveweight. Data were analysed from over 17,000 records from Merino and non-Merino ewe lambs from 22 different flocks across Australia. There were significant curvilinear relationships between liveweight or body condition score prior to breeding and reproductive rate for both Merino and non-Merino ewe lambs. When analysed together, there was a significant quadratic effect of body condition score on reproductive rate, independent of correlated changes in liveweight. The results indicated that if only a proportion of ewe lambs were selected for breeding, selection based on both liveweight and body condition scores may only improve overall reproductive rate by 1 to 2% compared to selection based on liveweight alone.

**Abstract:**

Ewe lambs that are heavier due to improved nutrition pre- and post-weaning achieve puberty at a younger age, are more fertile, and have a higher reproductive rate. Fatness is intimately linked to reproduction, and we hypothesised that higher body condition scores at breeding would have positive effects on the reproductive rate of ewe lambs over and above liveweight. We also expected that if only a proportion of ewe lambs were presented for breeding, then it would be more effective to select them based on both liveweight and body condition score. To test these hypotheses, we analysed data from over 17,000 records from Merino and non-Merino ewe lambs from 22 different flocks across Australia. Non-Merino ewe lambs were more fertile (69.4% vs. 48.7%) and achieved a higher reproductive rate than Merino ewe lambs (96.9% vs. 60.7%). There were significant curvilinear relationships between liveweight (*p* < 0.001) or body condition score (*p* < 0.001) prior to breeding and reproductive rate for both Merino and non-Merino ewe lambs. For both breeds, there was a significant (*p* < 0.001) quadratic effect of body condition score prior to breeding on reproductive rate, independent of the correlated changes in liveweight, and at the same liveweight, an extra 0.5 of a body condition score up to 3.3 improved reproductive rate by about 20%. Nevertheless, the results indicated that if only a proportion of ewe lambs were selected for breeding, then selection based on both liveweight and body condition scores may only improve the overall reproductive rate by 1 to 2% compared to selection based on liveweight alone. We conclude that liveweight is a more effective method than body condition score for selecting ewe lambs for breeding.

## 1. Introduction

Breeding ewe lambs at seven to 10 months of age can increase the profitability of sheep enterprises depending on their reproductive performance [1,2,3]. The reproductive performance of ewe lambs is highly variable, and multiple factors contribute to this variability, as reviewed by [4,5]. Merino ewe lambs that are heavier due to improved nutrition pre- and post-weaning consistently achieve puberty at a younger age, are more fertile (percentage of ewes pregnant per 100 ewes exposed to fertile rams), and have a higher reproductive rate (number of fetuses per 100 ewes exposed to fertile rams) [6,7,8,9]. This work using Merino ewe lambs with similar genetic background indicated that the relationship between liveweight prior to breeding and reproductive rate was linear over the range 35 to 50 kg and reproductive rate typically increased by about 4% per 1 kg in-crease in liveweight. Less is known about these responses for different genotypes of Merino or non-Merino ewe lambs managed in different environments. Thompson et al. [10] and Corner-Thomas et al. [11] analysed much larger datasets and reported curvilinear relationships between liveweight prior to breeding and reproductive rate for non-Merino ewe lambs which suggested little gains in reproductive rate from increasing liveweight above 45 to 50 kg. The relationship between liveweight and reproductive rate will influence the target liveweight at breeding for ewe lambs and potentially the minimum liveweight when deciding which ewe lambs are suitable for breeding.

Genetic factors influence the reproductive performance of ewe lambs [12,13,14] and are also likely to influence the average and minimum liveweight targets for ewe lambs at breeding. Puberty and fertility are intimately linked to body fatness, possibly via the actions of leptin [15,16,17,18,19], and in some studies, Merino ewe lambs with higher phenotypic or genetic fat depth measured at post-weaning age achieved a higher reproductive performance than those with lower fat depth [8]. More recently, Thompson et al. [9] reported that Merino ewe lambs from sires with higher Australian breeding values for fatness achieved a higher reproductive rate than ewe lambs from sires with lower values for fatness, and this effect was additive to the effects of liveweight prior to breeding. Indeed, Merino ewe lambs from genetically fatter sires could achieve comparable reproductive rates despite being mated lighter than those from genetically leaner sires, suggesting that ewe lambs with greater fat reserves are physiologically more mature at the same liveweight. Most commercial sheep producers in Australia do not have breeding values for individual animals, but there are strong phenotypic and genetic correlations between fat depth measured by ultrasound at the site used to generate breeding values for fat and body condition score at least in adult sheep [20,21]. We therefore expect that a higher body condition score will be positively related to the reproductive performance of ewe lambs. The effect of body condition score and liveweight are often confounded and difficult to separate, but we hypothesised that a higher body condition score at breeding would have an additional positive effect on the reproductive rate of ewe lambs over and above liveweight. It would also follow that if only a proportion of ewe lambs were selected for breeding, then it would be more effective to select these ewe lambs based on both liveweight and body condition score compared to liveweight alone.

## 2. Materials and Methods

### 2.1. Farms and Animals

**Figure 1:**
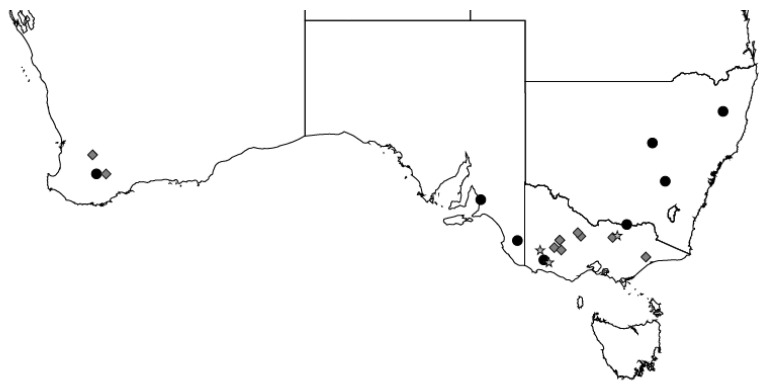
The locations of farms across southern Australia that provided data relating to the reproductive performance of ewe lambs for Merino (grey diamond), non-Merino (grey star), or both breeds (black circle).

The current study analysed data that were collected between 2009 and 2019 from 22 commercial and research farms across Australia. The data represented a range of production environments, sheep breeds, and genotypes within breeds (Figure 1). This included data from the Sheep CRC Information Nucleus Flock [22,23], MLA-funded Producer Demonstration Sites [24], and research or commercial flocks in Victoria (A. Kennedy, unpublished data) and Western Australia [6,7,8,9]. On average, each farm contributed data for three years, but this varied from one to eight years. The combined data included about 10,200 records for Merino ewe lambs and 7700 records for non-Merino ewe lambs, and all records were not available for some ewe lambs (Table 1). Dohnes and South African Mutton Merinos from the Information Nucleus Flock were categorised as Merinos. The non-Merino ewe lambs were either first-cross Merino x Border Leicester or Coopworth-based maternal composites.

Ewe lambs on all farms were managed under commercial conditions, and those from each farm were generally managed together from prior to breeding until at least pregnancy scanning. Ewe lambs were naturally mated between February and April, with rams for an average of 40-days (ranging from 21 to 74 days) using an average ewe/ram ratio of 50:1 (ranging from 25:1 to 100:1). Teasers (castrated rams or testosterone-treated castrated males) were used on four of the 22 farms for 15–17 days prior to the introduction of entire rams.

### 2.2. Data Collection

The liveweight of all ewe lambs was recorded at the start of breeding, and body condition score was assessed at the same time for some flocks or individuals within flocks. Body condition score was assessed using 0.2 to 0.3 increments by a single operator on each farm, using a one to five scale [25]. Pregnancy diagnosis at each farm was conducted by a commercial technician using transabdominal ultrasonography 50–60 days after the removal of entire rams. These data were used to calculate fertility and reproductive rate. 

### 2.3. Statistical Analysis

Merino and non-Merino ewe lamb data were analysed separately by the following methods using GENSTAT (Edition 19 [26]). The reproduction rate of individual ewes was analysed as various functions of different variables (separately or together), including liveweight and body condition score prior to breeding, using the method of generalised linear model with a multinomial distribution and logit link function and adjustments for location by year. For liveweight and body condition score prior to breeding, quadratic effects were also examined. Data from the eight separate flocks from the Information Nucleus Flock were combined since the ewe lambs at all sites were generated from common sires. All relevant effects were examined, and non-significant (*p* > 0.05) interactions were removed from the model. Liveweight was also examined by the method of restricted maximum likelihood for the effect of body condition score fitted as a fixed effect, while the year was fitted as a random effect.

## 3. Results

### 3.1. Liveweight and Body Condition Score at the Start of Breeding

The average liveweight and body condition score at the start of breeding varied widely between farms and years and between individual ewe lambs within farms. On average, non-Merino ewe lambs were slightly heavier (42.1 kg vs. 40.4 kg; Table 1) but with a similar body condition score prior to breeding compared to Merino ewe lambs (3.2 vs. 3.1; Table 1). The average standard deviation for liveweight and body condition score within farm and year was 5.2 kg and 0.27 units of a body condition score for Merino ewe lambs and 5.7 kg and 0.33 units of a body condition score for non-Merino ewe lambs. Across all ewe lambs, only 5.3% of Merinos and 11.2% of non-Merinos were greater than 50 kg, and only 4.6% and 9.2% were greater than body condition score 3.5 at the start of breeding.

**Table 1:** Summary of data collated on the reproductive performance of Merino and non-Merino ewe lambs from multiple flocks measured across one to eight years and mated at seven to eight and a half months of age. The data include the number of records and mean liveweight (kg) and body condition score at the start of breeding, and the number of records, average fertility (number of ewes pregnant per 100 ewes mated), and reproductive rate (number of foetuses scanned per 100 ewes mated) for each farm. The data for Flock 1 represent the average of eight separate sites. The data in brackets represent the 10th and 90th percentiles for liveweight and body condition score at the start of breeding for individual flocks.

Flock	Age	Liveweight		Body Condition Score		Pregnancy Scanning		
	(Months)	Records	Mean	Records	Mean	Records	Fertility	Reproductive Rate
Merino ewe lambs								
1	8.0	777	39.4 (30.6, 49.2)	764	3.0 (2.5, 3.5)	785	25.5	31.2
2	8.5	389	44.5 (38.5, 50.0)	389	3.1 (2.8, 3.4)	389	74.6	90.2
3	8.5	376	38.0 (30.5, 46.5)	376	3.1 (2.8, 3.3)	384	75.3	90.1
4	8.0	138	35.5 (32.0, 38.9)	192	2.8 (2.6, 3.1)	192	64.6	83.8
5	8.0	1581	42.0 (36.5, 47.5)	1475	3.3 (2.9, 3.7)	1592	57.5	70.7
6	7.0	999	38.2 (32.0, 45.0)	999	3.1 (2.8, 3.4)	999	63.7	81.2
7	7.5	444	39.2 (32.5, 45.5)	444	3.1 (2.8, 3.3)	444	14.0	17.0
8	8.5	680	33.9 (27.7, 41.3)	680	2.8 (2.5, 3.2)	680	20.1	22.1
9	8.0	712	44.9 (38.5, 51.5)	716	3.1 (2.8, 3.4)	719	56.3	73.0
10	8.0	1436	42.3 (34.5, 50.5)	355	3.3 (2.7, 3.7)	1466	47.1	56.3
11	8.0	2349	40.1 (34.3, 47.0)	593	3.1 (2.8, 3.4)	2543	47.9	61.7
Average	8.0	9881	40.4	6983	3.10	10,193	48.7	60.7
Non-Merino ewe lambs								
1	8	1366	42.7 (32.0, 52.4)	1142	3.1 (2.5, 3.8)	1452	43.5	56.0
12	8	1581	39.5 (33.5, 46.0)	1581	3.0 (2.5, 3.3)	1592	74.5	94.5
13	8	3149	43.8 (37.5, 51.0)	3151	3.3 (3.0, 3.7)	3151	83.8	125.9
14	7.5	1012	39.5 (33.0, 48.0)	1012	3.0 (2.7, 3.5)	1014	52.8	73.5
15	7.5	491	41.6 (35.0, 49.5)	491	3.2 (2.7, 3.5)	491	72.3	82.3
Average	7.8	7599	42.1	7377	3.17	7700	69.4	96.9

Across all farms and years, liveweight and body condition score at the start of breeding were moderately correlated for both Merino (slope = 8.8 ± 0.21 kg per body condition score; r^2^ = 25; *p* < 0.001) and non-Merino ewe lambs (slope = 9.2 ± 0.17 kg per body condition score; r^2^ = 0.31; *p* < 0.001). At a given liveweight, the upper and lower 90% confidence interval was 0.9 and 1.0 body condition score units either side of the mean for Merino and non-Merino ewes lambs, respectively.

### 3.2. Fertility and Reproductive Rate

Fertility and reproductive rate varied between farms by about 5-fold for Merino ewe lambs and 2-fold for non-Merino ewe lambs (Table 1). There was also considerable variation in fertility and reproductive rate between years within farm regardless of breed type. On average, non-Merino ewe lambs were more fertile (69.4% vs. 48.7%) and achieved a higher reproductive rate (96.9% vs. 60.7%) than Merino ewe lambs.

### 3.3. Effects of Liveweight or Body Condition Score at the Start of Breeding on Reproductive Rate

**Figure 2:**
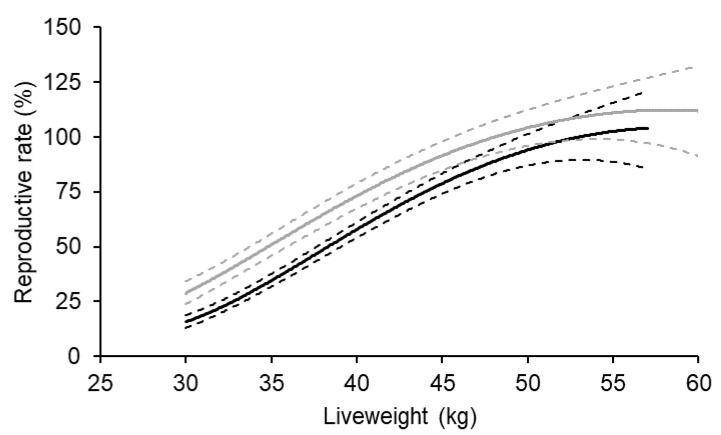
The effect of liveweight at the start of breeding at seven to eight and a half months of age on the reproductive rate (foetuses scanned per 100 ewes exposed to fertile rams) of Merino (black) and non-Merino (grey) ewe lambs. The Merino data represent 9881 records from 11 flocks, and the non-Merino data represent 7599 records from five flocks. The dotted lines represent the upper and lower 95% confidence intervals.

On average, there was a significant (*p* < 0.001) curvilinear relationship between liveweight at the start of breeding and reproductive rate for both Merino and non-Merino ewe lambs (Figure 2). The relationship with reproductive rate for both breed types were essentially linear up to about 50 kg, and a kilogram increase in liveweight between 30 and 50 kg was associated with a 3.9 and 4.7% increase in reproductive rate for Merino and non-Merino ewe lambs, respectively. If ewe lambs achieved 50 kg at the start of breeding, their reproductive rate was 90% and 98% of the predicted maximum for Merino and non-Merino ewe lambs, respectively.

**Figure 3:**
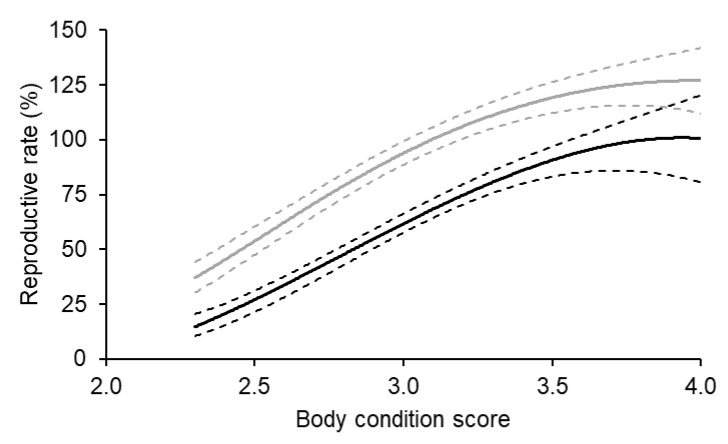
The effect of body condition score at the start of breeding at seven to eight and a half months of age on the reproductive rate (foetuses scanned per 100 ewes exposed to fertile rams) of Merino (black) and non-Merino (grey) ewe lambs. The Merino data represent 6983 records from 11 flocks, and the non-Merino data represent 7377 records from five flocks. The dotted lines represent the upper and lower 95% confidence intervals.

On average, the relationship between body condition score at the start of breeding and reproductive rate was also curvilinear for both Merino and non-Merino ewe lambs (Figure 3). The relationship with the reproductive rate for both breed types were essentially linear between body condition score 2.5 and 3.5, and over this range, the reproductive rate increased by 64% and 66% for Merino and non-Merino ewe lambs, respectively. If ewe lambs achieved a body condition score of 3.5 at the start of breeding, their reproductive rate was 90% and 96% of the predicted maximum for Merino and non-Merino ewe lambs, respectively.

### 3.4. Effects of Liveweight and Body Condition Score at the Start of Breeding on Reproductive Rate

**Figure 4:**
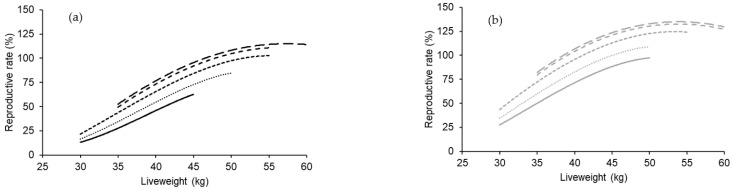
The effect of liveweight and body condition score at the start of breeding at seven to eight and a half months of age on the reproductive rate (foetuses scanned per 100 ewes exposed to fertile rams) of Merino (**a**) and non-Merino (**b**) ewe lambs. Within each figure, the body condition score responses are 2.5 (bottom line), 2.7, 3.0, 3.3, and 3.5 (top line). The Merino data represent 6919 records from 11 flocks, and the non-Merino data represent 7375 records from five flocks. The average 95% confidence intervals were ±4.9% for Merino ewe ewes and ±6.2% for non-Merino ewe lambs.

There was a significant quadratic effect of body condition score (*p* < 0.001) and liveweight (*p* < 0.001) at the start of breeding on reproductive rate (Figure 4). The effect of body condition score was independent of the correlated changes in liveweight, which was the dominant explanatory term. The effects of increasing body condition score at a given liveweight on the reproductive rate for both Merino and non-Merino ewe lambs were most evident below body condition score 3.3. When ewe lambs weighed more than 35 kg at the start of breeding, an extra 0.5 of a body condition score up to 3.3 improved the reproductive rate for both Merino and non-Merino ewe lambs by about 20%. There were no significant differences in reproductive rate between Merino or non-Merino ewe lambs that had a body condition score of 3.3 or more at the start of breeding after adjusting for liveweight.

### 3.5. Selecting Ewes for Breeding Based on Liveweight and Body Condition Score

As expected, if only a proportion of ewe lambs are selected for breeding, as the minimum liveweight increases fewer lambs are retained and the average liveweight, body condition score and predicted reproductive rate of those retained increases (Table 2). Above 35 kg, a one-kilogram increase in minimum liveweight would reduce the proportion of ewe lambs retained by about 6% and increase the average liveweight and predicted reproductive rate of those retained by about 0.6 kg and 1.9%, respectively.

To assess the effectiveness of selecting a proportion of ewe lambs for breeding based on liveweight and body condition score compared to liveweight alone, the reproductive rate of individual ewe lambs was also predicted based on both their liveweight and body condition score. When fewer lambs are selected for breeding based on achieving a liveweight threshold, there is more opportunity to identify ewe lambs from below the lightweight threshold but with a higher body condition score and predicted reproductive rate. The predicted reproductive rate of this cohort which maybe up to 2.5 kg lighter is typically 12 to 15% higher than for the lightest ewe lambs initially retained based on liveweight only. However, as selection based on liveweight and body condition score only allows for up to 20% of all ewe lambs to be substituted, the predicted improvement in overall reproductive rate compared to selection on liveweight alone was generally less than 2% across all the scenarios for both ewe breeds

## 4. Discussion

The reproductive rate of Merino and non-Merino ewe lambs bred at seven to 8.5 months of age was positively influenced by both liveweight and body condition score at the start of breeding. The average reproductive rate of Merino ewe lambs was lower than that of non-Merino ewe lambs, but the impact of increasing liveweight and body condition score at the start of breeding on reproductive rate was similar. Across all ewe lambs and regardless of breed, there was a linear effect of liveweight on reproductive rate up to about 50 kg, with smaller gains in reproductive rate with increasing liveweight above 50 kg. Similarly, there was a linear effect of body condition score on reproductive rate up to 3.3, with smaller gains above a body condition score of 3.3. Increasing both body condition score and liveweight at the start of breeding had positive additive effects on reproductive rate, which supported our first hypothesis. However, if only a proportion of ewe lambs were selected for breeding, it was predicted that selection based on both liveweight and body condition score typically only improved reproductive rate by 1 to 2% compared to selection based on liveweight alone. Our second hypothesis was therefore not supported, and we conclude that liveweight is a more effective method than body condition score for selecting ewe lambs for breeding.

Ewe lambs that were heavier at the start of breeding achieved a higher reproductive rate than lighter ewe lambs. A one-kilogram increase in liveweight at the start of breeding between 30 and 50 kg was associated with a 3.9 and 4.7% increase in reproductive rate for Merino and non-Merino ewe lambs, respectively. These increases in reproductive rate per kilogram of liveweight at the start of breeding were within the range we have previously reported for both Merino ewe lambs [6,7,8,9] and non-Merino ewes lambs [10]. However, the response to liveweight is greater than the 2.5% reported for maternal composite, Romney, and Coopworth ewe lambs by Corner-Thomas et al. [11], and the 3.1% for Border Leicester Merino ewe lambs reported by Paganoni et al. [27]. This variation in responses to liveweight at the start of breeding observed between individual flocks of Merino ewe lambs and Border Leicester Merino ewe lambs suggests that the response varies more between individual flocks within breeds than between breeds. Therefore, in practice, it would be beneficial for individual farmers to quantify the relationship between liveweight and reproductive rate for their specific ewe lambs and management settings.

This is the first study to report a curvilinear relationship between liveweight at the start of breeding and reproductive rate for Merino ewe lambs. This may not have been previously observed due to a limited number of animals above 50 kg in prior studies, whereas in the current study, almost 500 Merino ewe lambs (6%) and 850 non-Merino ewe lambs (11%) exceeded 50 kg at the start of breeding. Our study, in conjunction with the findings of Thompson et al. [10], demonstrate that regardless of breed there is a diminishing response in reproductive rate as liveweight increases beyond 50 kg. Moreover, Thompson et al. [9] found no significant effects of liveweight of Merino ewe lambs at the start of breeding on the birth weight or survival of their progeny. Likewise, Thompson et al. [10] observed no benefits in either survival of non-Merino ewe lambs or their progeny when ewe lambs were more than 50 kg at the start of breeding. These results suggest that the potential increase in weaning rates from both Merino and non-Merino ewe lambs weighing above 50 kg at the start of breeding are likely to be minimal. In practice, especially when providing supplements, it is therefore likely to be more cost-effective to differentially allocate feed to lighter ewe lambs over the months prior to breeding to increase the proportion of the flock above a minimum liveweight and therefore suitable for breeding.

Ewe lambs with a higher body condition score at the start of breeding achieved a higher reproductive rate than those with a lower condition score. The relationships for both Merino and Maternal ewe lambs were essentially linear between body condition scores of 2.5 and 3.5 and over this range reproductive rate increased by more than 60%. This effect of body condition score had not been reported previously for Merino ewe lambs and the magnitude of improvement was two to five-fold more than that reported for non-Merino ewe lambs [11,27]. In our study there were minimal gains in reproductive rate with increasing body condition score above 3.5, but less than 5% and 10% of Merino and non-Merino ewe lambs were above body condition score 3.5 at the start of breeding. Consistent with these findings, Corner Thomas et al. [11] reported that reproductive rate did not differ significantly between ewe lambs that had a body condition score of 3 or more at the start of breeding, whereas Paganoni et al. [27] reported linear relationships. The precise reasons for the differences between studies are not known but could reflect differences in sheep genotypes, subjectivity of body condition score assessments, size of datasets, or methods for data analysis. Nevertheless, if either Merino or non-Merino ewe lambs are bred between seven and eight and a half months of age, it is clear that they should be managed to be as fat as realistically feasible at the start of breeding to improve their reproductive rate.

Body condition score and liveweight at the start of breeding had an additive effect on reproductive rate. Below body condition score 3.3, an extra 0.5 of a body condition score improved reproductive rate for both Merino and non-Merino ewe lambs by about 20% at the same liveweight. These findings contrast with those of Paganoni et al. [27] who found no additional effects of body condition score on the reproductive performance of Border Leicester Merino ewe lambs in addition to that explained by liveweight. A possible explanation for the differences between studies is that Paganoni et al. [27] analysed data for single flocks and years comprising an average of 50 ewe lambs (range 40 to 112), whereas the current analysis was across flocks and years involving almost 7000 ewe lambs for each breed. These additional effects of body condition score are not surprising given that other studies have highlighted that a particular ratio of fat to lean mass is normally necessary to initiate puberty and for the maintenance of female reproductive ability across a range of species [15,16,17,18,19]. Rosales et al. [7,18] reported a linear relationship between leptin concentration and reproductive rate even after including liveweight at the start of breeding in statistical models, and other work reported a linear relationship between Merino ewe lambs’ own breeding value for fatness and leptin concentration [6,18]. This is also consistent with the positive effect of sire breeding value for fatness at post-weaning age on reproductive rate for Merino ewe lambs over and above the effects of liveweight at breeding reported by Thompson et al. [9]. A surprising result was the quadratic effect of condition score such that there were no significant gains in reproductive rate above condition score 3.3 at a given liveweight. This suggests not only a lower fatness threshold to stimulate puberty but also an upper threshold for improving reproductive rate. This would seem biologically sensible but further work is needed to confirm relationships with both phenotypic and genetic differences in fatness to inform both management and selection strategies to optimise reproductive rate in ewe lambs.

Reproductive rate was increased by selecting ewe lambs for breeding based on a higher minimum liveweight and there were minimal additional benefits from selecting these animals on both liveweight and body condition score. Breeding from only the heaviest 50% of ewe lambs increased the overall reproductive rate by about 20%. Current industry guidelines regarding minimum liveweights for breeding vary from 40 to 45 kg but to our knowledge there is little economic justification for these liveweight thresholds. In practice, the optimum proportion of ewes mated is likely to vary between enterprises. Young and Thompson [28] reported that the optimum proportion of ewe lambs to mate was consistently higher for non-Merino than Merino ewe lambs. This outcome is not surprising given our data which indicated that non-Merino ewe lambs were heavier at joining and achieved a higher reproductive rate at the same liveweight than Merino ewe lambs. It is also likely that other factors such as the length of the growing season, seasonal conditions that influence weaning weight and post-weaning growth, date of joining, value of meat, and costs of supplements will influence the optimum proportion of ewe lambs to breed in any given season. Furthermore, it is likely that the adoption of joining ewe lambs could be facilitated by the development of a decision support tool to identify management targets and priorities that reflect the seasonal conditions each year.

## 5. Conclusions

This study has shown that higher body condition scores at the start of breeding had additional positive effects on the reproductive rate of both Merino and non-Merino ewe lambs over and above correlated increases in liveweight. Reproductive rate was increased by selecting ewe lambs for breeding based on a higher minimum liveweight, and it is possible to identify ewe lambs from below the lightweight threshold but with a higher body condition score and predicted reproductive rate. However, it was predicted that selection for breeding based on both liveweight and body condition score only improved the overall reproductive rate by 1 to 2% compared to selection on liveweight alone.

## Figures and Tables

**Table 2:** The effects of selection based on minimum liveweight (kg) on the proportion of Merino and non-Merino ewe lambs retained for breeding (%) and their average liveweight (kg), body condition score, and predicted reproductive rate (foetuses scanned per 100 ewes exposed to fertile rams). The effects of replacing varying proportions of ewe lambs that were even lighter than a minimum liveweight threshold but with a higher body condition score and predicted reproductive rate on the overall predicted reproductive rate are also shown.

Ewe Lambs Retained for Breeding Selected on Liveweight Only					Ewe Lambs Retained for Breeding Selected on Liveweight and Body Condition Score		
Minimum Liveweight	Proportion Retained	Average Liveweight	Average Body Condition Score	Average Reproductive Rate	Proportion Replaced from Lighter Ewe Lambs	Minimum Liveweight	Average Reproductive Rate
Merino ewe lambs							
None	100	40.3	3.10	63.6	-	-	-
≥35.0	82	42.3	3.17	71.5	4.7	30.8	72.2
≥37.5	69	43.5	3.20	76.0	7.8	35.0	77.1
≥40.0	54	44.9	3.23	80.8	10.4	37.5	82.2
≥42.5	37	46.6	3.25	85.6	14.9	40.1	87.5
≥45.0	23	48.4	3.28	90.1	19.0	43.0	92.4
Non-Merino ewe lambs							
None	100	42.0	3.16	99.7	-	-	-
≥35.0	89	43.2	3.21	106.1	2.8	32.0	106.5
≥37.5	77	44.3	3.24	110.8	4.6	35.0	111.5
≥40.0	62	45.7	3.28	116.0	7.3	37.8	117.1
≥42.5	45	47.4	3.33	121.1	10.8	40.4	122.6
≥45.0	31	49.3	3.39	125.4	13.8	43.1	127.2

## Data Availability

The datasets generated and/or analyzed during the current study are not publicly available but are available from the corresponding author upon reasonable request, pending permission from project funders.
